# Temporo-Maxillary Articulation.—Abstract

**Published:** 1898-07

**Authors:** William Knight

**Affiliations:** Professor of Anatomy and Oral Surgery, Ohio College of Dental Surgery, Cincinnati, Ohio


### Temporo-Maxillary Articulation.—Abstract.

BY WILLIAM KNIGHT, M D., D.D.S., PROFESSOR OF ANATOMY AND ORAL SURGERY, OHIO COLLEGE OF DENTAL SURGERY, CINCINNATI, OHIO.

Read before the Section on Stomatology, American Medical Association, at Denver, Colorado, June 8, 1898.

### SOME REMARKS REGARDING VARIOUS AFFECTIONS OF THE TEMPORO-MAXILLARY ARTICULATION.

As introductory, it may be well to refer briefly to the anatomy of this articulation, so as to bring plainly before us its somewhat complicated construction.

The temporo-maxillary is a double arthrodial articulation, being possessed of two separate synovial membranes, that normally, in very rare instances, communicate with one another through an orifice in the inter-articular cartilage. Owing to this construction of the joint, the movements that take place in its inferior and superior compartments are of two kinds. In the upper the fibro-cartilage glides forward and backward in the glenoid cavity. In the lower compartment the condyle rotates on a transverso axis against the fibro-cartilage. When the mouth is widely opened these two movements are combined; the jaw and fibro-cartilage together move forward and rest upon the articular eminence, while at the same time the condyle revolves on the fibro-cartilage.

When the lower incisors are protruded beyond those of the upper jaw, the movement is confined chiefly to the upper articulation; whereas the lateral or grinding movement is accomplished more largely by the lower articulation. Owing to the anatomical arrangement of this joint, we occasionally see, as the result of disease in this articulation, instances in which partial impairment of its movements has occurred and in which this restricted mobility is more marked in the one than in the other of its compartments. Cases of that more distressing affection, viz: complete anchylosis of this joint, are, of course, much more frequently encountered by us in our practice than are those referred to above, and these would be still more frequent were it not for the anatomical construction of this joint, which, by virtue of its two compartments, offers a partial barrier against its easy invasion by disease.

Any cause that may produce disease or injury in the other articulations, will act with equal force upon this joint. From its more exposed position and from its occasional involvement from external ear diseases and dental inflammations, it is more liable to be affected than are the better protected articulations.

Trauma, unless it be of a slight degree, is more apt to result in complete, than in partial, anchylosis. Scarlet fever, diphtheria, measles, small pox, typhoid, typhus fever and tuberculosis are among those affections which have contributed to swell the list of cases of partial and complete anchylosis of the temporo-maxillary articulation. Rheumatic and gouty affections are much more likely to produce pain, stiffening and impairment of movement by working organic changes in one or more of the structures of the joint, than to lead to complete anchylosis.

Rheumatism more frequently attacks the cartilagineous and body structures of the joint, destroying, in many instances, the fibro-cartilage, and frequently causing atrophy or complete absorption of the condyle and portions of the glenoid cavity.

Cruveilier, who first described an example of rheumatoid arthritis of the temporo-maxillary articulation, says: "I have never seen the disease I call wearing away of the articular cartilage better marked than it was in this case. The condyle of the lower jaw did not exist. It might be supposed to have been sawed off horizontally at the line of junction of the head with the neck, and that which remained of the neck had been flattened. The articular part of the glenoid cavity was represented by a plain surface; no trace of inter-articular or cartilage of incrustation existed. Both surfaces of the altered articulation were remarkably red."—(*Anatom-Pathalogique*, LIV., lx).

The opposite condition of hypertrophy, as the result of rheumatism of this joint, has been recorded. This hypertrophy of the condyle and neck may occur in otherwise healthy individuals, and may produce sufficient displacement of the teeth so as to disarrange the normal bite. Heath, in his work on the diseases of the jaw, (third edition, page 420), gives an account, with illustrations, of an extreme case of this kind, in which the patient had a queer, lop-sided appearance of the face.

The milder results of this disease, as affecting the temporo-maxillary joint, is to produce that condition to which attention was first directed by Sir Astley Cooper, and described by him as "subluxation." He held the opinion that this condition depended upon relaxation of the ligaments of the joint. Heath, however, believes it is more frequently due to rheumatic changes in the articulation, and says: "The fact that these patients suffer more in damp weather and when the general health is feeble shows that it depends upon an arthritic diathesis, and the relief that is obtained by counter irritation and the administration of anti-rheumatic remedies proves that the complaint cannot be due to purely mechanical causes."

Gouty affections cause more acute pain and more or less acute swelling of the joint, accompanied or preceded by gastric disturbances. Repeated attacks of this disease are apt to result in permanent impairment of this joint by occasioning calcareous deposits among its structures, and this result is specially likely to occur in unrecognized or neglected cases of this nature.

Necrosis of the lower jaw originating from whatever cause (with the exception of exanthematous necrosis which is nearly always symmetrical and limited to the alveolar processes), may extend from any part of the bone to the articulation and seriously interfere with its mobility, or result in complete anchylosis.

Other affections originating in the neighboring soft parts, as scrofulous inflammation of the face, (John Howship) abscesses opening into the joint and the prolonged pressure of tumors, may each lead to partial or complete disorganization of this joint. As a a case illustrating the extension of a suppurative disease of the alveolar process to the temporo-maxillary joint, I refer to the following:

On August 9, 1897, I was asked by Dr. W. H. Creighton, of Cincinnati, to see a patient of his, Mrs. B., aged [illegible]5, who had suffered for five months past with an affection of the left side of the lower jaw. The disease had originated in the alveolus of the first molar on the affected side, giving rise to a necrosis of this process, which had gradually extended until the entire left arch of the bone had been invaded by the disease. On several occasions during the past few months Dr. Creighton had removed a number of fragments of necrosed bone, varying in size. At the time I saw the patient there was considerable tumefaction of the left side of the face, which extended from the lower border of the jaw as far upward as the zygoma. The soft tissues of the neck, reaching from the mastoid process downward along the anterior border of the sterno-mastoid muscle, were tense from inflammatory effusion. The parts involved were exceedingly sensitive to touch. An examination of the mouth brought into view a puffy, unhealthy line of gum tissue which extended from the incisor to the post-molar region, from which pus was seen to exude from several points. A flexible silver probe was, without much difficulty, passed into one of these pus openings, upward and outward, under the massater muscle, until it reached the condyle. At various points along this course the grating sensation of disintegrating bone was painfully felt. No operation, except one to enlarge the explored sinus, was recommended at this time. In addition to this, a lotion containing aconite, opium and stramonium was directed to be kept applied to the swollen cheek and neck, and the following prescription ordered:

℞ Tr. Aconite Rad., - - - - ʒss.

Potass. Acetatis, - - - - - ʒij.

Tr. Hyascyami, - - - - - ʒi.

Syrupi Capsici, - - - - - ʒiji.

Aqua q's, ad., - - - - - ℥iji.

Misce. ft. Misturae.

Sig Teaspoonful to be taken every two hours.

By this course of treatment the local swelling and pain of the parts was considerably relieved, and a threatening abscess under the chin was averted. In the course of a few days the diffused swelling, as at first seen, had become localized to the immediate neighborhood of the temporo-maxillary joint, where some fluctuation could now be detected. This was relieved by the use of the hypodermic syringe, and a considerable amount of pus was removed from the joint by the aid of this instrument. The fear now was that the case would terminate in anchylosis, and the apprehension as to such an unfortunate result was enhanced when, a few days after the operation, a piece of cartilage the size of a small finger nail was discharged from the sinus opening into the mouth. A careful examination of this specimen confirmed the belief that it was a fragment that had been cast off from the inter-articular cartilage.

From this time on the case progressed uneventfully, and at the present writing, May 10, 1898, an unexpectedly good result has been attained. The movements of the joint are but slightly impaired. The mouth can be opened nearly to its full extent, but in doing this the jaw is pulled slightly to the affected side.

The lateral or grinding movement is, however, more noticeably limited. The conclusion arrived at in this case is, that the fragment of cartilage discharged came from the lower compartment of the joint.

The treatment of this case, in addition to that given above, consisted in maintaining a free draining into the mouth and upon the surface. A wash for the mouth containing permanganate of potash was directed to be used frequently, and the sinuses were kept, as far as possible, in an aseptic condition by occasional injections of weak antiseptic solutions. Her general condition was improved by the internal administration of small doses of tartrate of iron and potash, and Fowler's solution of arsenic given in alternate doses. Both of these remedies are known to have a constructive power in many vitiated conditions of the blood. The former acts by entering into the composition of some of the proximate principles of the blood. The latter remedy probably acts by modifying the vital conditions of its organized constituents.

We know from clinical experience that these two remedies are of signal service in hastening, in many cases, the formation of healthy granulations around necrotic bone, reducing in this way to a minimum time the separation of the sequestrum, and aiding to bring many cases to a comparatively rapid cure. Owing to the somewhat unusual history and present termination of this case, it has been described in detail. The result so far appears to be an excellent illustration of a disease being limited to one compartment of the temporo-maxillary articulation, and although it is rather early to prognose as to the final outcome of the case, the conditions now are certainly favorable for a permanently good result.

The unhappy termination of comparatively slight injuries to the temporo-maxillary articulation in children can be best appreciated by bearing in mind the difference in the conditions of this joint in children and adults. Perhaps no writer has written more clearly and tersely upon this subject than has John Hilton in his work on "Rest and Pain," (2d edition, page 301), in which he says: "In adults the individual structures of a joint may be diseased and each may present its own local indications of special local symptoms. Thus we may meet with iso lated inflammations of the synovial membrane and ligaments, or a disease of the articular ends of bones in the adult.

"Now, although these structures are at all periods of life necessarily continuous with each other, and closely allied in function; yet it is at the adult period, after the completion of their development, that each separate structure seems to have acquired, and henceforth to manifest, both in health and disease, a structural iudependence which gives a character of individuality and isolation to the diseases of the different structures of the joint.

"In children all the structures of the joint must be formed, built up and nourished in concert, and in due relation to each other. On this intimate sympathy existing between the different parts of a joint during childhood, or during the period of growth, depends the tendency to diffuse disease contemporaneously in all the articular structures. Hence, we see in our practice the quick propagation of inflammation from one articulation to another, and a rapidity of implication of the various structures of the joint in childhood and youth, which we do not observe at a later period of life."

This tendency to a rapid and complete involvement of an injured joint in children should be constantly borne in mind by the surgeon when treating any articular disease in the youth. Especially should it be remembered that the discharge of pus from the ear in children is not unfrequently due to disease originating in the temporo-maxillary joint, and which is unfortunately at times not recognized until too late to prevent the disorganization of the articulation.

Affections to this articulation may occur at a very early period of life. Holt, in his work on "Infancy and Childhood," mentions a case of suppuration occuring in the temporo-maxillary joint at the early age of two weeks.

Acute arthritis in children is not a rare disease and is a suppurative one from the outset. As late results there may be pathological dislocation, or a flat joint; occasionally complete anchylosis. An early evacuation of pus in these cases may aid them to terminate favorably, but in neglected cases complete destruction of the joint often occurs. If more attention were given to early manisfestations of discomfort or of pain occuring in the temporo-maxillary articulation of infants and children, we would not be so frequently confronted by distressing cases of anchylosis of this joint.

TREATMENT.

The treatment of acute affections of the temporo-maxillary articulation should engage the early and watchful attention of the surgeon. All remedial means, local and constitutional, should be applied at once and persevered with until an obvious result has been obtained. Nor should his efforts be relaxed—neither should he become discouraged—even in those unpromising cases that apparently resist remedial measures, for by exercising patients he may bring to a successful termination a case that otherwise might result in complete anchylosis.

There can be no doubt that the local abstractions of blood in acute inflammatory diseases of joints, especially when this condition is due to trauma is one of our most potent means of preventing secondary changes in the structures of the articulations.

This local depletion can be best accomplished by the liberal use of the leech. Viz., twelve to twenty of these can be applied, according to the size of the affected joint and the amount of acute swelling present. For the temporo-maxillary articulation not more than four to eight are required. After their use bleeding should be encouraged by the application of hot fomentations. If leeches can not be secured, recourse can be had to the artificial leech or to the lancet. Internally arterial sedatives should be administered. The tartrate of potash and antimony, the mercurial compounds and aconite are remedies of recognized value in these cases. The successful surgeon will of course apply the general principles of treatment to each case committed to his charge, recognizing and meeting in each instance any special conditions of his patient that may in any way modify the local affection, whether this condition be of a temporary character, or one of a constitutional nature, as a rheumatic, tubercular, malarial or a specific taint of the system.

I cannot refrain from urging again the decided benefits to be obtained in the cases under consideration by the local abstraction of blood in the early stages of these affections. I am confident that many joints have been preserved by this treatment that would otherwise have become useless, and I desire to enter a protest against the growing neglect of local blood letting in acute synovitis, and in other acute inflammatory affections of the articulations.

In the sub-acute and in those cases of a more chronic nature in which complete anchylosis has not taken place, we can hope to obtain good results in most instances by the prolonged and judicious use of counter irritants, absorbants and mechanical devices. Iodine applied externally exerts a mild counter-irritant effect and also favors absorption of fibrinous exudate. Repeated blistering is of much service in many instances. The actual cautery applied, not directly over the joint, but in its near neighborhood, is an effective mode of treatment. Mercurial unguentums exercise, a curative effect, and although they have been extensively used in chronic joint affections, they have been made of more value by the preparation of oleates of this metal first introduced to the profession by John Marshall (surgeon of University College Hospital, London), who speaks highly of their utility in chronic inflammations, when the seat of the disease be in or sufficiently near the skin. He says: "I may first mention that not only persistent articular inflammation but also in simple synovitis, these remedies rapidly relieve the tenderness and pain and promote the absorption of the fluid effused into the joint. They are also of decided benefit in the rheumatic, the arthritic and the mixed forms of joint disease, but in these they do not supercede the necessity of general treatment."

He further states, "that these cleates of mercury should not be rubbed in like ordinary liniments or embrocations, but should be merely applied with a brush or be spread lightly over the part with one finger, otherwise they may cause cutaneous irritation or even produce a few pustules on the skin, especially in some persons."

After long use of these preparations I have become more and more convinced of their great usefullness in chronic inflammations of joints.

Among the mechanical means to be employed to prevent or overcome a gradual tendency to the closure of the jaws, due to disease of the temporo-maxillary joint, are the various forms of shields, wedges, gags that have been from time to time devised by dental surgeons, some of which have proved of service. Among these may be specially mentioned the device of Dr. Goodwillie, of New York, (archives of medicine, N. Y. June, 1881), who, writing of chronic cases of inflammation of the temporo-maxillary joint, says, "The method that I employ is as follows: In this case the patient is under the anæsthetic effect of morphine and nitrous oxide. If there is any rigidity of the muscles cautiously force open the mouth and take an impression of either the upper or lower teeth, and a rubber splint is made from the cast to cover all the teeth in one jaw. Upon the posterior part of this splint is made a prominence or fulcrum, so that when the mouth is closed the most posterior teeth close upon it while the anterior teeth are left free. The next step is to take a plaster of paris impression of the chin and from this make a splint. On each side of this splint is made a place for fastening elastic strips that pass up on each side of the head to a close fitting skull cap. When this appliance is in place and the elastic straps tightened so as to lift the chin, then pressure is brought to bear on the fulcrum at the posterior molar tooth, and so by these means extension is made at the joint and the inflamed surfaces within the joint are relieved from pressure, then immediate relief is experienced."

This method is an admirable one and is based upon sound surgical principles, for it secures to an inflamed part rest, which is a condition so essential to be obtained during the process of repair. In cases which have terminated in complete anchylosis, operative treatment is the only way to insure relief. In some few cases of fibrinous anchylosis, if a diagnosis can be made, it is practical to attempt the division of the fibrinous bands, and after this to make use of mechanical appliances in the effort to restore the mobility of the joint.

Mr. Spanton, of Harley (London, *Lancet*, April 16, 1881), published two cases of fibrinous anchylosis of the temporomaxillary articulation, in both of which we have proved the correctness of his diagnosis by dividing the fibrinous bands with a tenotome passed into the articulation. The patients were girls aged 9 and 10 respectively, and in both cases the disease of the temporo-maxillary joint had followed scarlet fever. Other surgeons have also reported instances of the same character.

It is difficult to make a diagnosis of fibrinous from bony anchylosis, but in a doubtful case the surgeon, before attempting a more serious operation, would be justified in using the tenotome to sever the fibrinous bands, and if successful in this, to forcibly open the mouth and effect a cure by these methods.

In all cases however, of bony anchylosis, the only relief to be obtained is by an operation upon the bone itself, either by resection of the condyle or by Esmarch's operation. The first operation for the removal of the condyle was by Professor Humphrey of Cambridge, (Associated Medical Journal, London 1856), and was undertaken for chronic rheumatic arthritis. Since that time resection of the condyle has been performed by many surgeons, and the operation has been generally adopted. This operation usually affords relief, provided the entire condyle be removed. A mere section of the neck or a partial removal of the condyle has proved to be inadequate. The operation is usually unattended with danger to life, but occasionally fatal results ensue, as in the following case: Miss M—— at the age of 4 years, fell from a porch striking upon her chin; pain and some swelling of both temporo-maxillary joints occured shortly after the accident followed by gradual persistent and increasing stiffness of movement in these joints, until finally, some nine months after the receipt of the injury complete anchylosis of the jaw became established. At the age of 19 years she was brought to me by Dr. C. A. Schuchardt of Cincinnati, as a patient for the Ohio College of Dental Surgery. Her appearance at this time was the usual one presented by the unfortunate subject of early and complete anchylosis of the temporo-maxillary joints. The marked dwarfishness of the chin owing to arrested developement was as marked as in any case ever under my observation, but she had experienced much less pain, and there was less irregularity and mal-position of the teeth than is usually in these cases. Her desire for an operation was mainly due to the fact that I had relieved a friend of hers who had been similarly afflicted from infancy and to whom I had given relief by performing a double Esmarch operation.

After an examination of the patient it was decided to resect the condyle. For this purpose she was placed in the Good Samaritan Hospital of this city, and the left condyle being the more enlarged was selected for the first operation. This condyle, which appeared to have become a part of the glenoid cavity, so firmly was it beded with the temporal bone, was removed in pieces. Three days after the operation the patient was in good condition and hopeful spirits. "I felt my jaw move on the other side," she said. On the fourth day, however, a secondary hemmorrhage occured. This was controlled by a gauze compress. Unfortunately, however, septic infection of a virulent form invaded the wound and shortly occasioned arteritis, that extended rapidly along the external carotid artery, which probably had been injured during the operation. A malignant cellulities of the adjoining parts occured and death resulted on the 12th day after the operation.

